# Vaccinating Animals

**Published:** 1881-10

**Authors:** 


VACCINATING ANIMALS.—Every veterinarian should make himself acquainted with the experiments and alleged discoveries of Pasteur regarding animal vaccination. We present an account of them elsewhere, in the Department of Progress of Veterinary Science. Prof. Pasteur claims to have discovered a method by which the virus of contagious diseases can be modified so as to secure to the animal immunity against disease. He professes to have done for some, if not all, of the contagious diseases of animals what Jenner did for small-pox. His claim is well substantiated in some directions, but it needs further proof and practical trial. Let us have some contributions from American veterinarians on this subject.

